# Commentary: Case report: Daratumumab treatment in pre-transplant alloimmunization and severe hemolytic anemia

**DOI:** 10.3389/fimmu.2023.1133382

**Published:** 2023-01-27

**Authors:** Jeremy W. Jacobs, Garrett S. Booth, Elizabeth S. Allen, Brian D. Adkins

**Affiliations:** ^1^ Department of Laboratory Medicine, Yale School of Medicine, New Haven, CT, United States; ^2^ Department of Pathology, Microbiology & Immunology, Vanderbilt University Medical Center, Nashville, TN, United States; ^3^ Department of Pathology, University of California San Diego, La Jolla, CA, United States; ^4^ Department of Pathology, University of Texas Southwestern Medical Center, Dallas, TX, United States

**Keywords:** daratumumab, alloimmunization, transfusion medicine, immunohematology, pre-transfusion testing, red blood cell antibodies, alloantibody, sickle cell disease

## Introduction

Pereda et al. recently reported three cases in which daratumumab was utilized to reduce the burden of red blood cell (RBC) and human leukocyte antigen (HLA) alloantibodies (1). These cases support the need for further investigation into the potential use of daratumumab and related plasma cell depletion therapies for patients with significant alloimmunization. However, the authors’ cases, particularly cases 1 and 2 in which the authors describe patients with sickle cell disease (SCD) and multiple RBC antibodies, lack sufficient immunohematology and transfusion details to reliably conclude the effectiveness and safety of daratumumab to reduce alloimmunization.

This therapeutic strategy, if indeed effective, would represent a significant advance in treating patients with high rates of RBC and/or HLA alloimmunization. However, the absence of details regarding blood transfusion compatibility and matching techniques employed, pre-transfusion testing and the specific methodology used, and patient RBC antigen status in these cases preclude the ability to definitively ascertain the effectiveness of daratumumab. We discuss issues that must be considered before considering this strategy as a viable option.

## Discussion

The primary issue at hand relates to the transfusion of patients with historic RBC antibodies. Modern transfusion practice requires antibody screens every three days for hospitalized patients, and blood banks maintain exhaustive records to prevent mis-transfusion in patients with historic antibodies. A negative antibody screen *via* routine pre-transfusion testing does not completely rule out the presence of alloantibodies, and perhaps more importantly, does not exclude the existence of plasma cells primed to produce antibodies directed against RBC antigens. While the authors state that all RBC antibodies were undetectable in patient 1, with three months of follow-up assessment reported, they do not discuss the testing employed. More concerning, the patient received four RBC transfusions following completion of daratumumab, the compatibility of which is not described. As most delayed hemolytic transfusion reactions (DHTRs) are due to an anamnestic response, it is worrisome to insinuate that antigen positive RBCs were provided because there was absence of an historic corresponding antibody based on testing. It is unclear if patient 2 was transfused after daratumumab therapy.

Plasma cells are notoriously hardy, and despite anti-plasma cell therapy, the ability to mount an antibody response may remain, especially as patients receiving daratumumab have been shown to form RBC alloantibodies (2). Also, suggesting that these alloantibodies are simply “negative”, especially without elaborating on the testing methodology by which they were not detected, is fraught with uncertainty, as different testing methods (e.g., tube testing, column agglutination, solid phase, or flow cytometry) have varying sensitivities, and some may be capable of detecting low-level antibodies while others cannot.

The effectiveness of daratumumab itself in these patients is unclear. While it does seem plausible that daratumumab was at least partially responsible for the disappearance of a portion of the alloantibodies, prior authors have shown that albeit uncommon, new antibodies can occur (2), and existing alloantibodies can persist despite daratumumab treatment (3–6). Secondly, the authors fail to acknowledge the possibility that the alloantibodies may have disappeared naturally through the phenomenon of evanescence. Numerous studies have shown that many patients’ RBC alloantibodies will decline in titer to levels below a detectable threshold without further exposure (7–9). Though the loss of all antibodies due to evanescence may be less likely, not accounting for this potential mechanism of a waning antibody is concerning.

Furthermore, literature demonstrates that even if these antibodies have become undetectable, these patients may still be at risk for DHTRs (10–13). Both patient 1 and 2 had histories of DHTRs, though their descriptions lack pertinent details. DHTRs occur when a transfused unit stimulates an immune response with an antibody directed at RBCs, either anamnestic or *de novo*, and can range in severity from a positive direct anti-human globulin test (DAT) to clinically significant hemolysis. Therefore, antibody identification, DAT, elution studies, and antigen status of the RBC donor unit are crucial for the diagnosis and prevention of DHTR (14, 15). As DHTRs can precipitate destruction of the patient’s intrinsic RBCs as well in a potentially fatal process known as hyperhemolysis, it is important not to induce these reactions, especially as they occur most frequently in patients with SCD. For patients with SCD, many institutions also provide units that are matched to the patient’s extended RBC phenotype to help prevent antibody formation (5). In a patient with a history of multiple DHTRs, it is essential to prevent further sensitization or additional reactions given the risk of hyperhemolysis. Significant cost and effort are undertaken to obtain antigen-matched, compatible blood for these patients, and the ability to premedicate with daratumumab would represent a substantial change in this fundamental practice.

In summary, we caution readers considering daratumumab or other antibody-depleting therapies for patients with significant RBC alloimmunization burden, and we would like to highlight the importance of a full assessment of RBC donor-recipient compatibility, thorough immunohematology testing, perhaps including a monocyte monolayer assay in certain cases, and careful planning with transfusion medicine professionals (16). It is imperative that this use of daratumumab is carefully evaluated for both safety and efficacy, preferably in murine models or using thoughtfully planned prospective clinical trials that appropriately balance benefit and risk. While Pereda et al. demonstrate a novel application of daratumumab for patients with extensive RBC alloimmunization, current standard of care requires transfusion honoring historic RBC antibodies. Nonetheless, exploration of the potential for this, and other molecules, to impact alloimmunization is warranted.

## Author contributions

JJ, GB, EA, and BA drafted and revised the manuscript. All authors approved the final version.
